# Factors influencing the latency of simple reaction time

**DOI:** 10.3389/fnhum.2015.00131

**Published:** 2015-03-26

**Authors:** David L. Woods, John M. Wyma, E. William Yund, Timothy J. Herron, Bruce Reed

**Affiliations:** ^1^Human Cognitive Neurophysiology Laboratory, Veterans Affairs Northern California Health Care System, MartinezCA, USA; ^2^The Department of Neurology, University of California Sacramento, DavisCA, USA; ^3^Center for Neurosciences, University of California Davis, DavisCA, USA; ^4^Center for Mind and Brain, University of California Davis, DavisCA, USA; ^5^Alzheimer’s Disease Center, Department of Neurology, University of California Davis, DavisCA, USA

**Keywords:** gender, timing, processing speed, motor, foreperiod, handedness, hemisphere, replication

## Abstract

Simple reaction time (SRT), the minimal time needed to respond to a stimulus, is a basic measure of processing speed. SRTs were first measured by Francis Galton in the 19th century, who reported visual SRT latencies below 190 ms in young subjects. However, recent large-scale studies have reported substantially increased SRT latencies that differ markedly in different laboratories, in part due to timing delays introduced by the computer hardware and software used for SRT measurement. We developed a calibrated and temporally precise SRT test to analyze the factors that influence SRT latencies in a paradigm where visual stimuli were presented to the left or right hemifield at varying stimulus onset asynchronies (SOAs). Experiment 1 examined a community sample of 1469 subjects ranging in age from 18 to 65. Mean SRT latencies were short (231, 213 ms when corrected for hardware delays) and increased significantly with age (0.55 ms/year), but were unaffected by sex or education. As in previous studies, SRTs were prolonged at shorter SOAs and were slightly faster for stimuli presented in the visual field contralateral to the responding hand. Stimulus detection time (SDT) was estimated by subtracting movement initiation time, measured in a speeded finger tapping test, from SRTs. SDT latencies averaged 131 ms and were unaffected by age. Experiment 2 tested 189 subjects ranging in age from 18 to 82 years in a different laboratory using a larger range of SOAs. Both SRTs and SDTs were slightly prolonged (by 7 ms). SRT latencies increased with age while SDT latencies remained stable. Precise computer-based measurements of SRT latencies show that processing speed is as fast in contemporary populations as in the Victorian era, and that age-related increases in SRT latencies are due primarily to slowed motor output.

## Introduction

Simple reaction time (SRT) tests, where subjects simply respond as fast as possible to the occurrence of a stimulus, are among the most basic measures of processing speed. SRTs were first studied by Francis Galton in the late 19th century (Johnson et al., 1985). More recent studies have shown significant correlations between SRT latencies of processing speed and measures of fluid intelligence (Deary et al., 2001; Sheppard and Vernon, 2008). Indeed, Jensen (2011) argued that SRT latencies provide one of the most objective metrics for comparing processing speed, and hence fluid intelligence, across different populations.

In a recent historical meta-analysis, Silverman (2010) found that SRT latencies have increased substantially since the Victorian era. For example, in studies performed from 1884 to 1893, Francis Galton recorded visual SRT latencies that ranged from 181 to 189 ms in subjects ranging in age from 18 to 60 years (Johnson et al., 1985). These latencies are considerably shorter than those reported in recent SRT studies (Lowe and Rabbitt, 1998; Deary et al., 2001; Deary and Der, 2005; Der and Deary, 2006). Given the correlation between SRTs and fluid intelligence (Deary et al., 2001; Bugg et al., 2006), Woodley et al. (2013) concluded that the slowed SRTs in recent studies reflected a systematic reduction in processing speed, and hence fluid intelligence, in contemporary populations.

However, an alternative explanation of the apparent SRT slowing is that the SRT latencies reported in recent studies have been inflated by hardware and software delays in computer-based paradigms (Dordonova and Dordonov, 2013). In support of this argument, contemporary studies using mechanical SRT measurements (Montare, 2010; Eckner et al., 2011), including SRT testing procedures similar to those used by Galton (Dordonova and Dordonov, 2013), report SRT latencies similar to those observed in the Victorian era.

This line of reasoning implies that SRT latencies have been consistently overestimated in computer-based studies performed over the past several decades. **Table 1** provides a summary of recent large-scale SRT studies: none of the contemporary, computer-based studies reported SRT latencies as short as those reported by Galton. However, mean SRT latencies reported in recent studies vary widely (**Table 1**), ranging from 233 ms (Krieg et al., 2001; Vincent et al., 2012) to nearly 400 ms (Bugg et al., 2006). These variations suggest that the magnitude of SRT overestimation may vary as a function of the paradigm and the computer system used for testing. One possible explanation for these variations is variable timing delays introduced by computer hardware and software that can increase measured SRT latencies by up to 100 ms (Neath et al., 2011). Therefore, in the current experiments, we used carefully calibrated computer hardware and high-precision computer software (see methods) that provided accurate computer-based SRT latency measurements, enabling corrections for hardware and software delays.

**Table 1:** Studies of age-related changes in visual simple reaction time (SRT).

Study	N	Age range	SRT (ms)	SD (ms)	IS-SD (ms)	CV (%)	Age slope	SOA	No. trials
Krieg et al. (2001)	4,896	20–59	233	96.5			0.34	2–5 s	40 (10)
Bugg et al. (2006)	196	20–89	397	142					32 (0)
Deary and Der (2005)	1,930	16–63	328	90	84	26%	1.70	1–3 s	20 (8)
Deary et al. (2001)	900	55	358	120	91	26%		1–3 s	20 (8)
Deary et al. (2011) box	150	18–80	256	38	50	20%	0.80	1–3 s	20 (8)
Deary et al. (2011) PC	150	18–80	274	49	45	16%	1.00	1–3 s	20 (8)
Dykiert et al. (2012b)	312	18–59	275	41	54	20%	0.50	1–3 s	20 (8)
Reeves et al. (2006)	2,261	17–46	285	67	94	33%	1.10	0.5–1.8 s	25
Vincent et al. (2008)	5,247	18–51	267	74			1.20	1–2 s	40 × 2
Vincent et al. (2012)	107,413	17–65	261	47			0.52	1–2 s	40 × 2
Experiment 1	1,469	18–65	231	27	40	17%	0.55	1–1.8 s	120 (20)
Experiment 2	189	18–82	238	28	53	22%	0.45	1–2 s	100 (20)

N, number of participants; SD, intersubject standard deviation; IS-SD, intrasubject (trial-to-trial) standard deviation; CV, coefficient of variation (IS-SD/SRT); Age-slope, estimated rate of increase in SRTs in ms/year; SOA, range of stimulus onset asynchrony; No. trials, number of trials, with the number of practice trials in parentheses.

The improved computer-based paradigm was used to analyze the effects of factors that have been found to significantly influence SRTs, including age, sex, and education. Previous studies have uniformly found significant age-related increases in SRT latencies (Wilkinson and Allison, 1989; Fozard et al., 1994; Inui, 1997; Anstey et al., 2005; Commodari and Guarnera, 2008; Deary et al., 2010; Godefroy et al., 2010; Era et al., 2011; Dykiert et al., 2012a; see **Table 1**). Increasing age has also been associated with increases in trial-to-trial SRT variance (Dykiert et al., 2012a; Bielak et al., 2014). However, the nature of age-related increases in SRTs remains incompletely understood. Increasing age could influence SRTs at two possible processing stages: (1) older subjects could take longer to detect a stimulus, and (2) older subjects could take longer to produce a response once a stimulus has been detected. In the current experiments, we were able to divide the SRT into stimulus detection time (SDT), the time needed to perceive the stimulus, and movement initiation time (MIT), the time needed to depress the response button, by subtracting an independent measure of MIT from the SRT of each subject.

Previous studies have also reported significant SRT differences between the sexes, with men generally showing shorter-latency SRTs than women (Krieg et al., 2001; Anstey et al., 2005; Era et al., 2011; Dykiert et al., 2012b; Vincent et al., 2012), although these effects are smaller and less consistent than age effects (Sheppard and Vernon, 2008). In addition, subjects with increased education generally have shorter-latency SRTs (Krieg et al., 2001; Anstey et al., 2005), although such effects are small or absent in some populations (Vincent et al., 2012; Ritchie et al., 2013).

We also analyzed the effects of two stimulus variables; preceding stimulus onset asynchrony (SOA) and the hemifield of presentation. When stimuli are presented at varying intervals, SRT latencies are strongly influenced by the preceding SOA, with shorter-latency SRTs obtained for stimuli delivered at longer SOAs (Niemi and Naatanen, 1981). Some studies have also suggested that these foreperiod effects are altered in older subjects (Bherer and Belleville, 2004; Vallesi et al., 2009), while other studies find similar effects in subjects of different ages (Greenwood et al., 1993).

Simple reaction time latencies to stimuli presented to the left and right hemifield also vary: SRTs are slightly faster to stimuli that are delivered to the visual field contralateral to the hand used in responding, which directly activates the hemisphere controlling motor responses, and therefore avoids the additional delay associated with callosal transmission (Clarke and Zaidel, 1989; Bisiacchi et al., 1994; Brizzolara et al., 1994; Chaumillon et al., 2014). Some studies have also suggested that aging has differential effects on the two cerebral hemispheres (Berlingeri et al., 2013), with greater age-related declines in right than left hemisphere function (Benwell et al., 2014), with some studies reporting disproportionate age-related SRT latency increases to stimuli presented in the left visual field (Robinson and Kertzman, 1990).

We analyzed the effects of age, education, sex, handedness, SOA, and hemifield of presentation on SRTs in two large-scale, computer-based experiments incorporating precise timing control. Experiment 1 examined SRTs in a population sample of 1469 New Zealand adults ranging in age from 18 to 65 years. Experiment 2 examined an independent sample of 189 California subjects ranging in age from 18 to 82 years.

## Methods: Experiment 1

**FIGURE 1:**
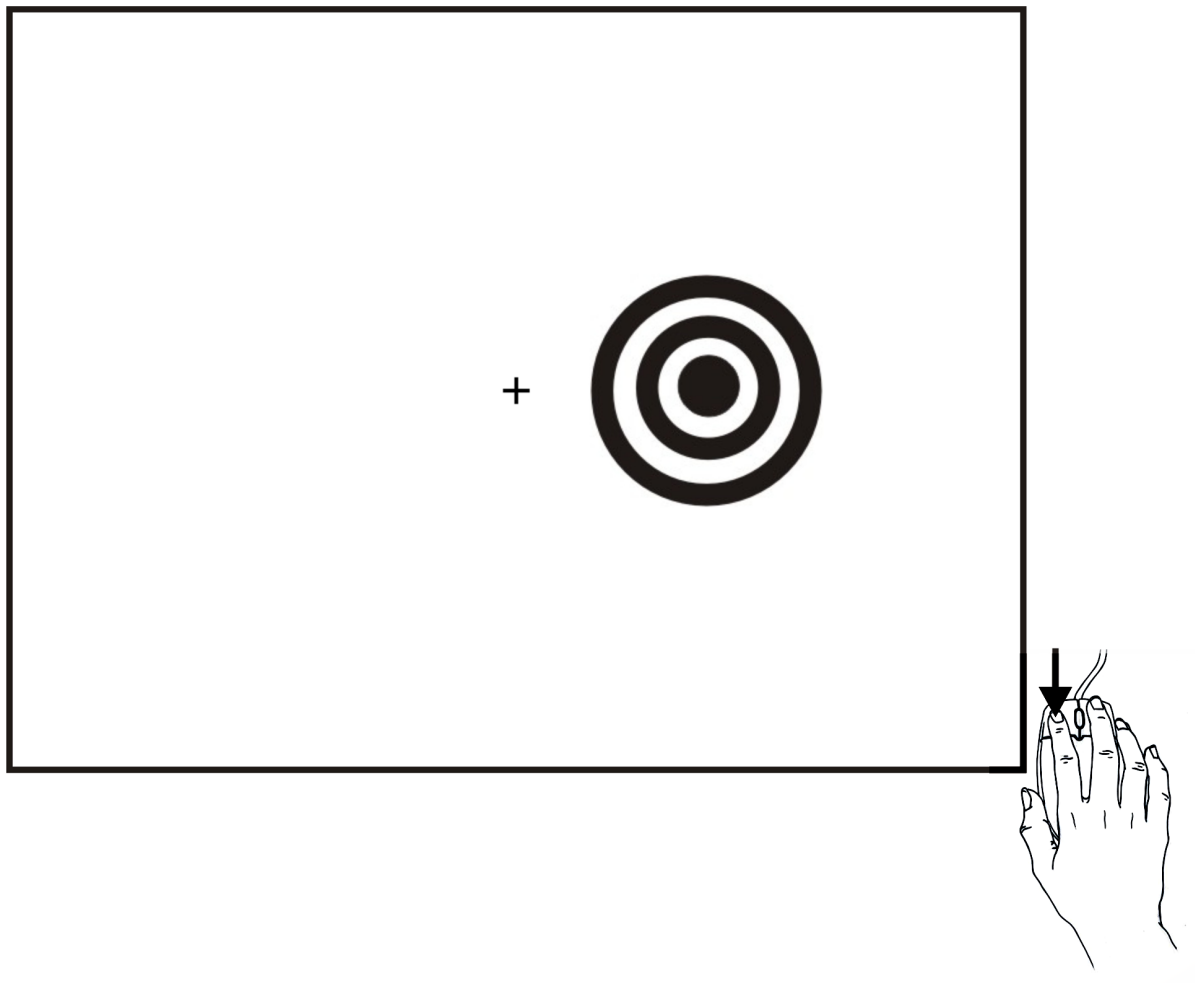
**Stimuli and task.** Stimuli were high-contrast bulls eyes presented to the left or right hemifield for a duration of 200 ms at randomized stimulus onset asynchronies (SOAs) that ranged from 1000 to 1800 ms in five 200 ms steps in Experiment 1, and from 1000 to 2000 ms in five 250 ms steps in Experiment 2. Stimuli could occur in the visual hemifield ipsilateral (shown) or contralateral to the responding hand. Subjects responded to all stimuli as rapidly as possible by depressing the mouse button with the index finger of their dominant hand (i.e., right-handed subjects depressed the left mouse button and left headed subjects depressed the right mouse button).

As shown in **Figure 1**, subjects responded as rapidly as possible to stimuli presented to the left or right hemifield by depressing a response button with the index finger of their dominant hand. The task was designed to elicit SRTs with short latencies, and incorporated a number of design features to assure precise SRT measurement: (1) The response button was a computer gaming mouse designed for ultrafast responding with minimum force, displacement, and timing uncertainty; (2) Stimuli were large and of high luminance and contrast; (3) SRT windowing functions excluded response latencies less than 110 ms and greater than 1000 ms; (4) Twenty practice trials were given to each subject, and SRTs were gathered from 120 test trials; (5) Computer hardware and software delays were measured.

### Participants

We studied a subset of 1637 community volunteers in Rotorua, New Zealand, who had participated in a study of the neuropsychological and health effects of environmental exposure to varying levels of naturally occurring hydrogen sulfide (H_2_S; Reed et al., 2014). Because we wanted to compare performance across several neuropsychological tests, we eliminated 75 subjects who lacked complete data sets in the finger tapping (Hubel et al., 2013a), SRT, or choice reaction time (CRT) tests (Woods et al., 2015). We also eliminated 41 ambidextrous subjects whose finger tapping data had not been analyzed, 33 subjects who failed to respond consistently to some types of stimuli in a separate CRT task, and 19 subjects with unexplained poor accuracy on the SRT test (the mean SRT hit-rate for these subjects was 65%).

Of the remaining 1469 participants, 40.1% were men, 10.8% were left-handed by self-report (based on writing hand), and all were between 18 and 65 years of age (mean age = 46.3 years. for men, 45.4 years. for women). Participants had an average U.S. equivalent of 12.6 years of education, including 77.1% who had secondary school qualification, 48.4% of whom had a qualification beyond secondary school, such as a bachelor’s degree (12.2%), master’s degree (3.0%), doctorate (1.6%), or other trade, technical, or professional qualification (31.6%). Most subjects were of European background (80.0%) or New Zealand Maori (15.5%), and 78.8% were employed. Institutional Review Board approvals for study procedures were obtained at the University of California, Berkeley and from the Northern Ethics Committee in New Zealand. Prior written informed consent was obtained from all participants. Subjects wore prescription lenses as required.

### Apparatus and Stimuli

Simple reaction times were recorded as part of a 30 min. cognitive assessment that included four other tests from the California Cognitive Assessment Battery (CCAB): finger tapping (Hubel et al., 2013a,b), CRT in a visual feature conjunction task (Woods et al., 2015), digit span (Woods et al., 2011a,b), and an adaptive Paced Auditory Serial Addition test. Testing was performed in a quiet room using a PC controlled by Presentation software (Versions 13 and 14, NeuroBehavioral Systems, Albany, CA, USA). Participants practiced for 20 trials before the 120 test trials began, and sat 0.7 m from a 17^′′^ Samsung Syncmaster LCD monitor, whose refresh rate was 60 Hz. The SRT test and instructions are available online^1^.

**Figure 1** shows the stimulus, a black-and-white bull’s eye subtending 4° of visual angle. Stimuli were presented randomly and equiprobably to the left and right hemifield, 3.6° from a central fixation cross that remained illuminated. Because SRT latencies are influenced by stimulus contrast (Pins and Bonnet, 1996; Ratcliff and Van Dongen, 2011) and brightness (Ratcliff and Van Dongen, 2011; Parker, 2014), stimuli were presented on a bright background (40 cd/m^2^) and were of high contrast (dark rings were 0.16 cd/m^2^). Stimulus durations were fixed at 200 ms. Five different SOAs were used, ranging randomly from 1000 to 1800 ms in equiprobable, 200 ms steps. Overall, 24 stimuli were presented at each SOA, half to the left and half to the right hemifield. SRT testing required ∼4 min.

### Timing Calibration: Hardware and Software Delays

The precision with which reaction times are collected depends on the computer hardware and software used for measurement (Plant and Turner, 2009). There are two principal sources of hardware delay, which aggregately can inflate true SRT values by up to 100 ms (Neath et al., 2011). First, there is a delay in the actual appearance of the stimulus after the computer video card sends the stimulus image to the LCD monitor, which depends on monitor electronics. We measured the delay for the Samsung Syncmaster monitor with a photodiode (StimTracker, Cedrus, San Pedro, CA, USA) and found a mean delay of 11.0 ms (SD = 0.1 ms). Second, there is a variable delay between the moment that the response button is depressed and the moment that the response is registered by the device driver and detected by the computer software controlling the experiment. For a USB response device, the delay depends on the device design and the device driver software that signals the event to the operating system, and by the frequency with which the stimulus-delivery software polls the driver to determine if a response has occurred. While standard mouse drivers may introduce delays of 20 ms or more before registering a response, software engineers attempt to minimize such delays when designing mice for computer-gaming applications by shortening the movement required for button closure and writing device drivers with high USB sampling rates. In the current experiment, we used a PC gaming mouse (Razer Sidewinder, Carlsbad, CA, USA) that required minimal movement (2mm) for button closure and interfaced with a device driver with a 1.0 kHz USB sampling rate. We measured response delays by disassembling the mouse and simulating button closure with an electronic relay. The average response delay was 6.8 ms (SD = 1.8 ms). Thus, total delays introduced by the video display and mouse averaged 17.8 ms.

In addition to hardware delays, software interruptions can introduce unpredictable delays that may increase SRT latencies and latency variability. The frequency and duration of software interruptions depends on both the design of the stimulus-delivery software and the number and type of extraneous software processes concurrently running on the computer. If a response occurs during an interruption (i.e., when the stimulus-delivery programming has been temporally halted), the occurrence of the response will nevertheless be captured by the response driver, but the latency of the response will not be calculated by the stimulus-delivery program until it returns to execution and samples the response device. These timing interruptions must be continuously monitored throughout an experiment to assure timing precision. Presentation software reports event-time uncertainties for each event during an experiment by continuously sampling the 100 kHz programmable clock. When an extraneous software process interrupts the experiment, there is a corresponding gap in the otherwise continuous event-timing record, and an event occurring during the gap will show a corresponding increase in event-time uncertainty. For example, if a response occurred during a software interruption lasting 10 ms, its latency would be reported as having occurred immediately after the gap, but the associated event-time uncertainty would be 10+ ms. In the current experiment, the PC was configured to minimize extraneous software interruptions. The analysis of the event-time uncertainties for all (264,566) events that occurred during the experiment showed that software interruptions had a minimal influence on measured SRT latencies: the median event-time uncertainty was 0.1 ms, with 99.9% of events showing event-time uncertainties of less than 1.05 ms.

### Data Analysis

A response window of 110–1000 ms was used. Responses outside this range were categorized as false alarms (FAs). The failure to respond during the 110–1000 ms interval following a stimulus was categorized as a miss. Hit rate was defined as the percentage of stimuli associated with valid responses. For each subject, hit-rate, false-alarm rate, and mean SRT latency were calculated along with intrasubject (trial-to-trial) SRT variance.

### Statistical Analysis

Participants were classified into seven different 7-year wide age ranges (e.g., from 18–24 years to 59–65 years). The results were first analyzed using a multifactor mixed ANOVA with Age-Group, Sex, SOA, and Hemifield (ipsilateral or contralateral to the preferred responding hand) as independent variables. Separate ANOVAs were performed for mean SRT, hit rate, intrasubject SRT SD, and intrasubject coefficient of variation. Greenhouse-Geisser corrections of degrees of freedom were uniformly used in computing *p* values in order to correct for any non-spherical covariation within factors or interactions. Effect sizes are reported as ω^2^ values. Correlation analysis was also used to analyze the effects of age, education, sex, and handedness on SRTs, and to develop age-regression functions. When correlations were significant, a 95% confidence interval range was calculated with SPSS. Certain pairwise effects were analyzed with Student’s *t*-test, using a model that assumes unequal variance in the different subject groups when appropriate.

## Results: Experiment 1

**FIGURE 2:**
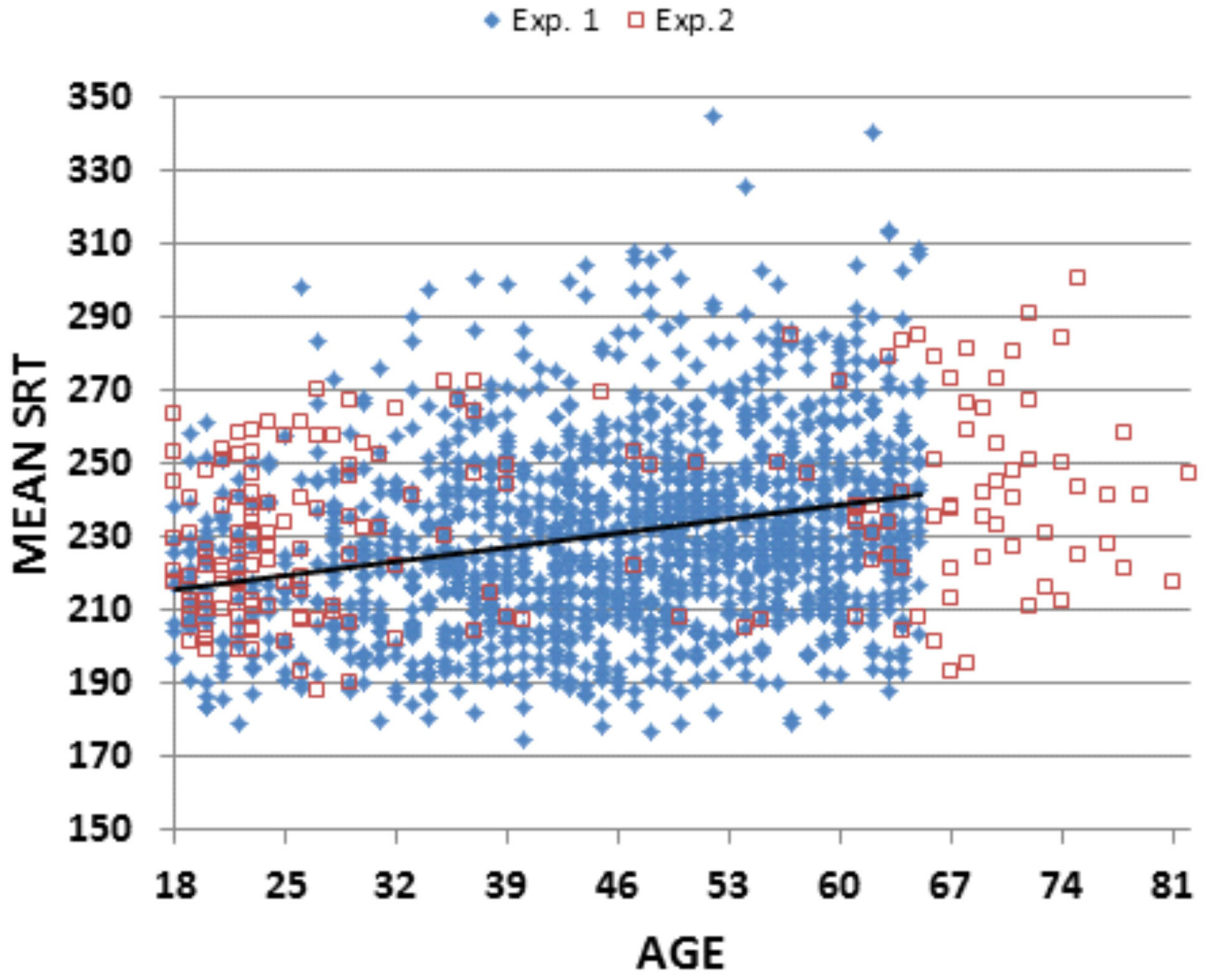
**Simple reaction times (SRTs) as a function of age.** From subjects in Experiment 1 (blue diamonds) and Experiment 2 (open red squares). Eight subjects from Experiment 1 with mean SRTs above 350 ms are not shown. The linear trend line is from Experiment 1 data.

**Figure 2** shows Experiment 1 SRT latencies (blue diamonds) as a function of age, and **Table 2** shows the different measures for each of the seven age groups and for the entire experiment. An ANOVA for repeated measures showed a significant effect of Age-Group on SRT [*F*(6,1462) = 15.52, *p* < 0.0002, ω^2^ = 0.06]. SRT latencies were shorter than those seen for the other studies in **Table 1**. SRT latencies increased from 217.8 ms (200 ms when latencies were corrected for hardware delays) in the youngest subject group, to 239.1 ms (222.3 ms, delay-corrected) in the oldest subject group. However, the effect size of age was relatively small: power analysis showed that a 99% probability of detecting a significant (*p* < 0.05) effect of age would require 458 subjects. Age-related slowing occurred throughout the age range, with significant (*p* < 0.05, uncorrected) pairwise differences seen between Group 1 (G1) and G3–G7, between G2 and G4–G7, between G3 and G5–G7, between G4 and G5–G7, and between G6 and G7.

**Table 2:** Age-related changes in performance.

Age range	G1: 18–24	G2: 25–31	G3: 32–38	G4: 39–45	G5: 46–51	G6: 51–58	G7: 59–65	Experiment 1	Experiment 2
N	86	115	201	273	276	272	246	1469	189
Mean age	20.8	28.6	35.5	42.3	48.6	55.0	61.6	45.8	41.0
% male	34%	36%	42%	37%	41%	44%	40%	40%	58%
SRT	217.9	221.0	224.8	227.7	233.6	236.4	239.1	230.8	237.8
SRT SD	19.5	22.8	23.4	26.6	27.2	27.0	28.1	26.8	27.8
IS-SD	39.4	37.2	37.1	39.1	41.4	40.2	43.2	40.0	52.7
CV	18%	17%	16%	17%	18%	17%	18%	17.1%	21.9%
Hit-rate	93.9%	96.7%	97.2%	97.2%	97.6%	97.6%	97.4%	97.1%	97.2%
VF-D	6.76	6.97	7.84	7.63	8.40	8.30	7.16	7.74	10.5
SDT	125.8	131.8	134.0	132.6	133.5	130.2	127.7	131.2	138.3
SOA-D	30.6	25.7	27.0	29.8	27.9	27.3	29.6	28.3	26.9

VF-D, absolute difference in SRTs for stimuli in the left and right visual fields; SDT, stimulus detection time (the difference between SRT and movement initiation time measured in a finger tapping task); SOA-D, difference in SRTs following short (1000 ms) and long (1800 ms) SOAs. Physical calibration suggests that “true SRTs” are reduced by 18 ms and SDTs by 11 ms. See ****Table 1**** for descriptions of additional labels.

**Table 3** shows the correlation matrix for Experiment 1. Regression analysis showed significant correlations between age and SRT latencies [*r* = 0.24 (range 0.19–0.29), *t*(1469) = 9.47, *p* < 0.0001], which showed an age-slope of 0.55 ms/year. Intraindividual SRT SDs (mean 40.0 ms) also showed an effect of Age-Group [*F*(6,1462) = 4.72, *p* < 0.03, ω^2^ = 0.02], and increased weakly with age [*r* = 0.11 (range 0.06–17), *t*(1469) = 4.24, *p* < 0.0001]. However, when intrasubject SRT variance was normalized by each individual’s SRT latency, the resulting intraindividual coefficient of variation (CV, mean 17.14%) did not change significantly with age [*r* = 0.04].

**Table 3:** Correlation matrix for Experiment 1.

	Edu	SRT	Hit-rate	AR-SRT	CV	SDT	VF-D	SOA-D	MIT
Age	0.01	0.24	0.17	0.00	0.04	-0.02	0.03	0.01	0.33
Edu		-0.05	0.09	-0.05	-0.06	0.01	-0.06	-0.06	-0.08
SRT			0.25	0.97	0.31	0.71	0.20	0.26	0.25
Hit-rate				0.21	-0.26	0.22	-0.10	-0.08	0.00
AR-SRT					0.31	0.73	0.20	0.27	0.17
CV						0.19	0.19	0.16	0.12
SDT							0.03	0.01	-0.51
VF-D								0.07	0.05
SOA-D									0.05

Given the sample size (*N* = 1466), correlations exceeding |*r*| = 0.07 are statistically significant with Bonferroni correction (*p* < 0.002). MIT, movement initiation time. AR-SRT, age-regressed SRT latency *z*-score, using the age-regression function from Experiment 2. See **Tables 1** and **2** for descriptions of additional labels.

Hit-rate (mean 97.1%) was also affected by Age-Group [*F*(6,1462) = 16.85, *p* < 0.0001, ω^2^ = 0.06], and there was a significant correlation between age and hit-rate [*r* = 0.17 (range 0.12–0.22), *t*(1469) = 6.61, *p* < 0.0001]. Both effects were due to reduced hit-rates in the youngest subject group (93.9%) compared to the other groups (mean 97.2%), without significant differences between any of the other groups. Hit-rate also correlated with SRT latency [*r* = 0.25 (range 0.20–0.30), *t*(1467) = 9.89, *p* < 0.0001]; i.e., slower subjects were slightly more accurate. Multiple regression showed that both age and hit-rate were independently associated with SRT latency [age, *t*(1466) = 8.27, *p* < 0.0001; hit-rate, *t*(1466) = -8.38, *p* < 0.0001].

Subjects made an average of 3.87 FAs (3.2% of responses). The FA distribution was highly skewed (median = 1.71%, skew = 3.68), with 52.1% of subjects committing fewer than 2% FAs, and 5.7% of subjects producing more than 10% FAs. There was a strong negative correlation between hit-rate and FA-rate [*r* = -0.83, *t*(1467) = -57.00, *p* < 0.0001]: i.e., subjects who made more FAs missed more targets. In addition, increased FA rates were associated with shorter SRT latencies [*r* = -0.25, *t*(1467) = -9.89, *p* < 0.0001] and increased SRT variance [*r* = 0.27, *t*(1467) = 10.74, *p* < 0.0001]. This likely reflects the occurrence of occasional anticipatory responses within the SRT window, producing very short-latency hits that would both reduce the mean SRT and increase mean SRT variance. Finally, younger subjects (who had lower hit-rates) made more FAs, producing a negative correlation between age and FA-rate [*r* = -0.14, *t*(1467) = -5.42, *p* < 0.0001].

We examined fatigue effects by comparing SRTs over successive blocks of 20 trials (e.g., 1–20, 21–40, etc.). SRTs increased from 228.1 ms in the initial 20-trial block, to 237.2 ms in the final 20-trial block, producing a significant fatigue effect with small effect size [*F*(5,8165) = 88.22, *p* < 0.0001, ω^2^ = 0.05]. The fatigue effect did not correlate with age [*r* = -0.01], or with any other demographic variable.

Subjects were slightly faster (by 0.61 ms) when responding to stimuli presented in the visual field contralateral to the responding hand [*F*(1,1468) = 5.23, *p* < 0.05 ω^2^ < 0.01]. This small effect was not significantly affected by age [*r* = -0.05, *t*(1467) = -1.92, *p* < 0.06]. The mean difference between SRT latencies to stimuli presented in the left vs. right visual field was 0.56 ms. This difference was marginally reduced with age [*r* = -0.06, *t*(1467) = 2.30, *p* < 0.03]; i.e., this effect was opposite the prediction that older subjects would show a greater increase in slowing for stimuli presented to the left visual field. The absolute value of the difference in SRT latencies to stimuli in the two visual fields was also small (7.74 ms) and did not vary with age [*r* = 0.03] or sex [*r* = 0.01].

Simple reaction times did not differ significantly between male and female subjects [*F*(1,1467) = 2.83, *p* < 0.10], nor were significant sex differences seen in trial-to-trial SRT variance [*r* = -0.01] or CVs [*r* = 0.01]. Education did not significantly influence SRT latencies [*r* = -0.05, *t*(1467) = 1.92, *p* < 0.06], but was weakly associated with increases in hit-rate [*r* = 0.09, *t*(1467) = 3.46, *p* < 0.001], reduced SRT variance [*r* = -0.07, *t*(1467) = 2.69, *p* < 0.01], and reduced CVs [*r* = -0.06, *t*(1467) = 2.30, *p* < 0.03]. Handedness did not influence SRTs [*r* = -0.02], SRT variance [*r* = -0.04], or SRT CVs [*r* = -0.03].

**FIGURE 3:**
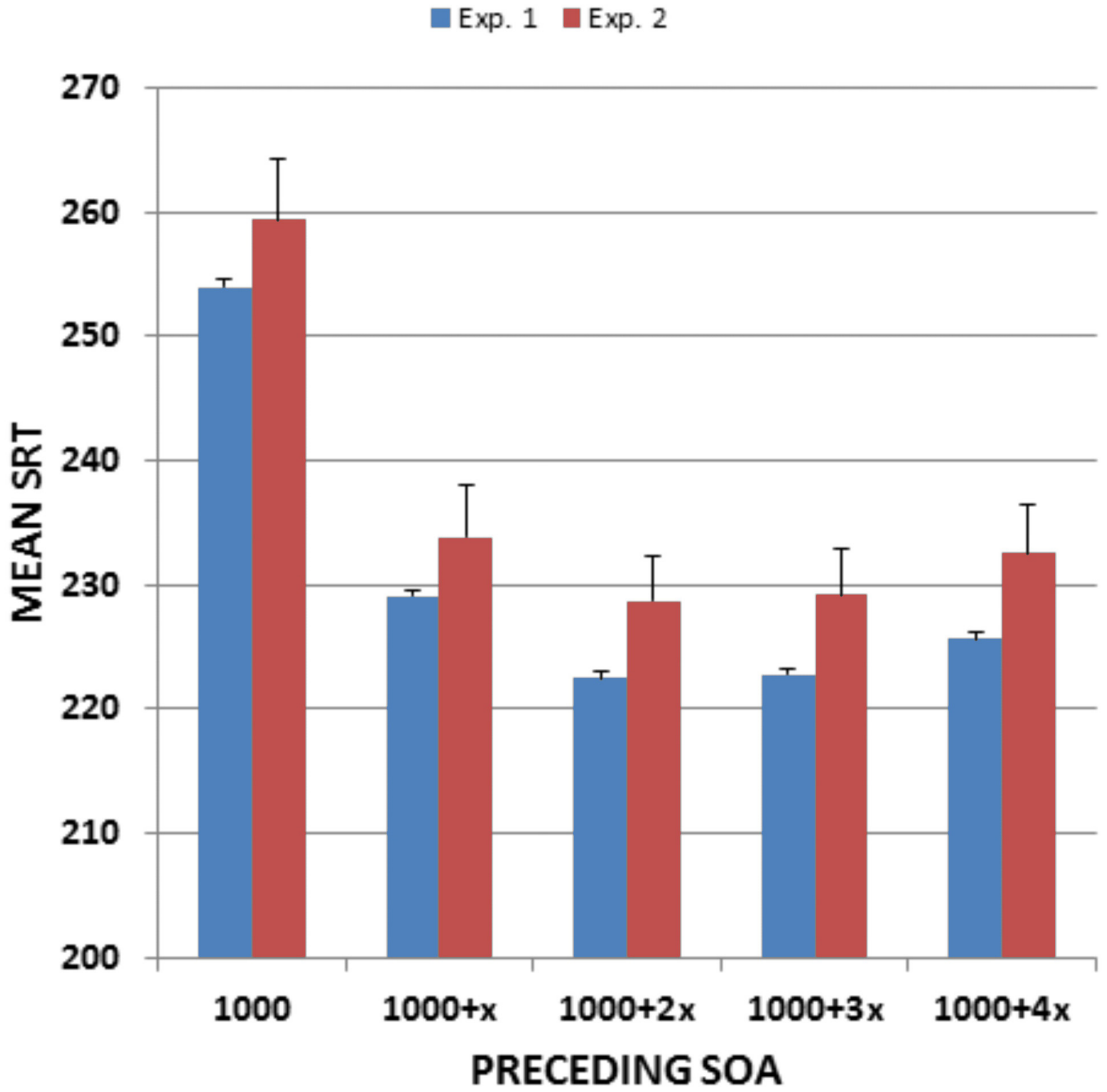
**Mean SRTs as a function of preceding SOA.** From Experiment 1 and Experiment 2. Error bars show 95% confidence intervals. *X*, SOA step size (200 ms in Experiment 1 and 250 ms in Experiment 2).

Stimulus onset asynchronies had a highly significant effect on SRTs [*F*(4,5848) = 1419.79, *p* < 0.0001, ω^2^ = 0.49], as shown in **Figure 3**. SRTs were prolonged (by roughly 15%) at the shortest SOA. The SOA effect size was large, and power analysis indicated that a 99% probability of detecting a significant (*p* < 0.05) effect of SOA would require only 10 subjects. Age did not alter SOA effects, with the Age-Group × SOA interaction failing to reach significance [*F*(24,5848) = 2.09, *p* < 0.06]. RT variance also increased at the shortest SOA [*F*(4,5848) = 126.47, *p* < 0.0001, ω^2^ = 0.08], again without a significant Age-Group × SOA interaction.

**FIGURE 4:**
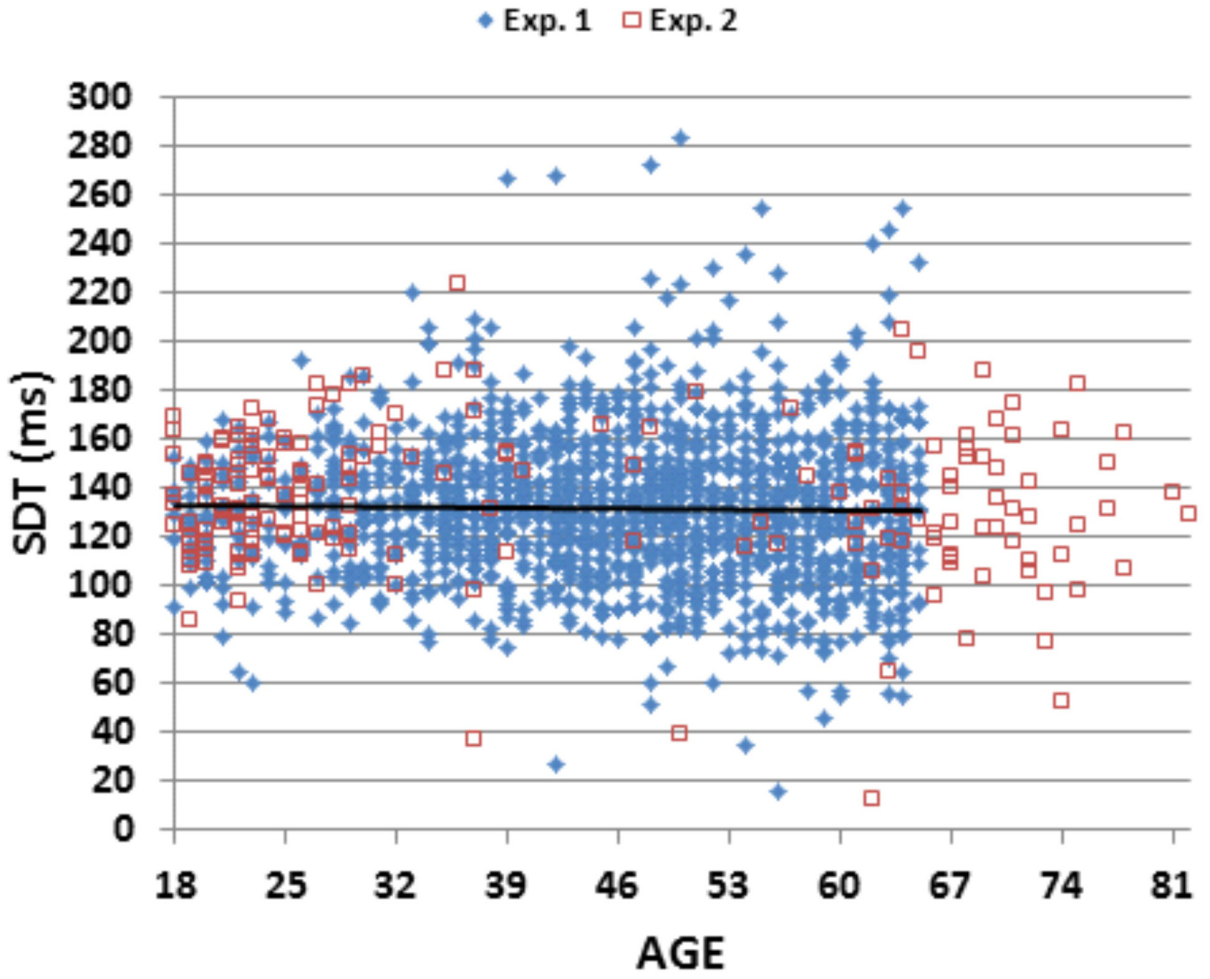
**Stimulus detection time (SDT) as a function of age.** SDT was derived by subtracting the movement initiation time in a speeded finger tapping test from SRTs. The linear trend line is from Experiment 1 data.

We found a significant correlation [*r* = 0.25 (range 0.20–0.30), *t*(1,1469) = 9.89, *p* < 0.0001] between SRT latencies measured in the current experiment and MIT, the time needed to depress the response button that had been previously measured in a self-paced finger tapping task conducted on the same day (Hubel et al., 2013a). SDT latencies, obtained by subtracting MIT latencies from SRT latencies, averaged 131.2 ms (SD = 30.2 ms). **Figure 4** shows SDT latencies as a function of age. Unlike SRT latencies, SDT latencies did not increase with age [*r* = -0.02], nor did they differ significantly between male and female subjects [*r* = 0.05]. Finally, comparisons of the correlations of age with MIT latency and age with SRT latency showed significantly larger correlations of age with MIT latency [*r* = 0.33 vs. *r* = 0.24, *z* = 2.65, *p* < 0.01].

## Discussion: Experiment 1

The mean SRT latencies of 231 ms obtained in the current study were substantially shorter than those reported in most previous computerized SRT studies (**Table 1**). When corrected for the hardware delays associated with the video display and mouse response (17.8 ms), “true” SRTs in Experiment 1 ranged from 200 ms in the youngest subject group to 222 ms in the oldest, i.e., 15–30 ms above the SRT latencies reported by Galton for subjects of similar age (Johnson et al., 1985). However, based on Galton’s notebooks, Dordonova and Dordonov (2013) argued that Galton recorded the shortest-latency SRT obtained out of three independent trials per subject. Assuming a trial-to-trial SRT variance of 50 ms (see **Table 1**), Galton’s reported single-trial SRT latencies would be 35–43 ms below the mean SRT latencies predicted for the same subjects; i.e., the mean SRT latencies observed in Experiment 1 would be slightly less than the mean SRT latencies predicted for Galton’s subjects. Therefore, in contrast to the suggestions of Woodley et al. (2013), we found no evidence of slowed processing speed in contemporary populations.

Simple reaction time SDs were substantially reduced in comparison to those of previous studies, ranging from 18% to 55% of the variances reported in **Table 1**. Intrasubject (trial-to-trial) SRT variance was also reduced, ranging from 44% to 69% of the values reported in previous studies. Within- and between-subject variance measures were likely reduced by the increased precision of SRT measurement, as well as by the number of practice trials, the number of test trials, and the windowing functions used for SRT measurement.

### Aging Effects

Mean SRTs increased with age, with significant differences seen between most adjacent age groups. The age-regression slope of 0.55 ms/year fell within the range of SRT/age slopes reported in previous studies (range 0.34–1.7 ms/year, see **Table 1**). In addition, we found that the youngest subject group made more errors, consistent with suggestions that older subjects are generally more conservative than younger subjects (Forstmann et al., 2011). However, a multiple regression analysis indicated that SRTs showed a highly significant influence of age, even after the influence of hit-rate had been factored out^2^.

Stimulus detection times averaged 131 ms and did not change with age, suggesting that the rate of accumulation of sensory information (Miller and Ulrich, 2003) was not age-sensitive. The results are consistent with previous modeling studies which show that older subjects accumulate sensory information as rapidly as young subjects, but are delayed in responding (Ratcliff et al., 2001). Interestingly, SDT latencies (120 ms after correcting for display delays in the monitor) were similar to the latencies of early cortical components of the visual evoked potential (Yordanova et al., 2004), which, like SDTs, appear to have stable latencies across the adult life span (Emmerson-Hanover et al., 1994).

Other studies have also suggested that aging effects on SRT latencies are largely the result of slowed motor output. For example, previous electrophysiological studies have found age-related reductions in corticospinal excitability (Levin et al., 2011). In addition, in an SRT task where subjects were required to lift their finger and press a target button following the appearance of a stimulus, Era et al. (2011) found larger age-related changes in the movement phase than in the detection phase. These results imply that SRT tasks that place greater demands on motor responding, for example, by using response buttons that require greater force or displacement for button closure, may enhance the apparent magnitude of age-related slowing, thus contributing to the variability of the age-slopes seen in **Table 1**.

As in previous studies (Deary and Der, 2005; Der and Deary, 2006; Dykiert et al., 2012a), we found significant increases in SRT variance with age. However, we found no significant age-related changes in the CV, which remained considerably smaller than those reported in previous studies, even in our oldest subject group. One possible explanation is that older subjects require more training to become familiar with the SRT task. As a result, brief tests with limited pre-test training may disproportionately increase variance in older subjects.

### SOA and Hemifield Effects

Stimulus onset asynchrony effects did not differ with age, suggesting that temporal expectancy effects are preserved during normal aging. This finding contrasts with some previous reports (Bherer and Belleville, 2004; Vallesi et al., 2009), but is consistent with others (Greenwood et al., 1993).

Both visual-field asymmetries and the absolute value of visual field asymmetries were small and not age-sensitive. SRT latencies showed only a small reduction (<1 ms) for stimuli presented in the hemifield contralateral to the responding hand, as reported in previous studies (Chaumillon et al., 2014). The latency difference was considerably less than the hypothesized latency (10+ ms) required for transcallosal transmission (Caminiti et al., 2009). The small magnitude of the contralateral advantage suggests that visual SRTs may depend on bilateral visual representations at cortical or subcortical levels, rather than on unilateral cortical stimulus processing and transcallosal communication.

### Sex Differences

We found no significant differences between male and female subjects in SRT latencies, SRT variance, CVs, SOA effects, or hemifield effects. A number of previous large-scale studies have found longer SRT latencies in female subjects, along with increased variance and CVs (Fittro et al., 1992; Fozard et al., 1994; Anstey et al., 2005; Dykiert et al., 2012b; Vincent et al., 2012). However, other studies have failed to find significant sex differences (Annett and Annett, 1979; Gottsdanker, 1982; Der and Deary, 2006).

As with aging effects, sex differences may be sensitive to response demands, such as the force or distance needed for button closure. It is well established that the speed of finger tapping is reduced in female subjects, due primarily to an increase in the time that the response button is held down (Hubel et al., 2013a). In a recent study, Era et al. (2011) found larger sex differences in the movement phase than in the detection phase of reaction time studies. Thus, SRT studies that minimize the difficulty of button closure may also reduce sex differences. In addition, sex differences are reportedly reduced with increased familiarity with the task, and may disappear with more extensive practice (Reimers and Maylor, 2006).

## Experiment 2: A Replication

Previous large-scale SRT studies using apparently similar paradigms and subjects have widely varying SRT latencies (**Table 1**). For example, two studies performed by the same laboratory (Deary et al., 2001, 2011) reported mean SRT latencies that differed by more than 80 ms. Since the mean SRT latencies in Experiment 1 were less than the latencies of most previous studies, a replication of the results in a separate population was needed to evaluate generalizability. Therefore, in Experiment 2, we compared the results of Experiment 1 with the results from a separate population of 189 subjects ranging in age from 18 to 82 years, tested on a different continent.

## Methods: Experiment 2

The methods of Experiment 2 were similar to those in Experiment 1, with two minor modifications. First, SOAs were increased in steps of 250 ms rather than 200 ms, so that SOAs ranged from 1000 to 2000 ms in Experiment 2 rather than 1000 to 1800 ms, as in Experiment 1. Second, the SRT test included 100 rather than 120 test trials, while the number of practice trials (20) remained the same.

### Participants

We studied 189 subjects in Experiment 2, whose demographic characteristics are summarized in **Table 2**. Subjects were recruited from advertisements in the San Francisco Bay Area on Craigslist and from pre-existing control populations, and underwent testing with the entire California Cognitive Assessment Battery (CCAB)^3^. Subjects were required to meet the following inclusion criteria: (a) fluency in the English language; (b) no current or prior history of psychiatric illness; (c) no current substance abuse; (d) no concurrent history of neurologic disease known to affect cognitive functioning; (e) on a stable dosage of any required medication; (f) auditory functioning sufficient to understanding normal conversational speech and visual acuity normal or corrected to 20*/*40 or better. Subject ethnicities were 64% Caucasian, 12% African American, 14% Asian, 10% Hispanic*/*Latino, 2% Hawaiian*/*Pacific Islander, 2% American Indian*/*Alaskan Native, and 4% “other.”

All subjects signed written consent forms approved by the institutional review board (IRB) at the Veterans Affairs Northern California Health Care System (VANCHCS), and were compensated for their participation. Unlike the subjects in Experiment 1, who had been recruited as a community sample with balanced age distributions, the age distribution of subjects in Experiment 2 was bimodal: 104 subjects were below the age of 35 years, 24 subjects were between the ages of 35 and 59 years, and 61 subjects were between the ages of 60 and 82 years. The subjects were slightly younger, on average, than those in Experiment 1 [*t*(1656) = 4.70, *p* < 0.0001], and slightly better educated [*t*(1656) = 8.64, *p* < 0.0001], with an average of 14.6 years of education. Experiment 2 subjects were predominantly male (58% vs. 40% in Experiment 1). The 45 subjects over the age of 65 years were particularly well-educated (15.1 years of education). In order to make comparisons between the results of Experiments 1 and 2 with different subject age distributions, we used the age-regression equation from Experiment 1 and calculated *z*-scores in both experiments based on Experiment 1 values.

Identical computer hardware and software were used in the two testing laboratories so that the measured hardware delays were identical to those in Experiment 1. However, because the test computer in Experiment 2 was disconnected from the network, timing uncertainties due to operating system interruptions were reduced compared to those of Experiment 1: median event-time uncertainties for 62,400 events averaged 0.1 ms (range 0.1–32.0 ms), with more than 99.9% of events showing event-time uncertainties of less than 0.3 ms.

## Results: Experiment 2

The results of Experiment 2 are summarized in **Table 2**, and data from Experiment 2 (red squares) are included in **Figures 2**, **3**, and **4**. Mean SRTs averaged 237.8 ms, 7 ms greater than the mean SRTs of Experiment 1. Applying the age-regression function from Experiment 1 revealed that the SRTs in Experiment 2 were 9.7 ms above predicted values, producing a small, but significant difference in age-corrected SRT latencies between the two experiments [*F*(1,1656) = 23.24, *p* < 0.0001, ω^2^ = 0.01]. In contrast, hit-rates were virtually identical in the two experiments (97.1 vs. 97.2%).

The correlation matrix for Experiment 2 is provided in **Table 4**. As in Experiment 1, there was a significant correlation between age and SRTs [*r* = 0.35 (range 0.21–0.48), *t*(187) = 5.11, *p* < 0.0001], reflecting an age-slope of 0.45 ms/year. There was also a significant correlation between age and inter-trial SD [*r* = 0.33 (range 0.20–0.47), *t*(187) = 4.78, *p* < 0.0001]. However, unlike Experiment 1, there was a significant correlation between age and the CV [*r* = 0.27 (range 0.13–0.41), *t*(187) = 3.84, *p* < 0.0002].

**Table 4:** Correlation matrix for Experiment 2.

	Edu	SRT	Hit-rate	AR-SRT	CV	SDT	D-VF	D-SOA	MIT
Age	0.16	0.35	-0.19	-0.08	0.27	-0.07	0.05	-0.05	0.43
Edu		-0.06	-0.10	-0.13	-0.11	0.01	-0.03	-0.02	-0.06
SRT			-0.07	0.91	0.35	0.58	-0.02	0.15	0.31
Hit-rate				0.01	-0.44	0.09	-0.19	0.08	-0.17
AR-SRT					0.25	0.65	0.30	0.18	0.14
CV						0.02	0.12	0.50	0.32
SDT							0.12	-0.04	-0.60
DVF								-0.19	-0.07
D-SOA									0.17

Given the sample size (*N* = 189), correlations exceeding |*r*| = 0.20 are statistically significant with Bonferroni correction (*p* < 0.002). See previous tables for descriptions of additional labels.

As in Experiment 1, education had no significant effect on SRT latency [*r* = -0.06, *t*(185) = -0.82, NS], nor were there significant effects of education on hit-rate, intrasubject SRT latency variance, CVs, or SDTs. Similarly, there were no significant sex differences in SRT latencies, intrasubject SRT variance, CV, or SDTs. However, there was a significant sex difference in hit-rate in Experiment 2: female subjects had slightly higher hit rates than men [*r* = -0.23 (range -0.09 to -0.37), *t*(187) = 3.23, *p* < 0.002].

**Figure 3** shows the effects of preceding SOAs on SRTs. SOA effects were similar to those seen in Experiment 1 [*F*(4,752) = 125.22, *p* < 0.0001, ω^2^ = 0.40], with SRT latencies reduced by 26.9 ms at the longest compared to the shortest SOA (versus 28.3 ms in Experiment 1). As in Experiment 1, SOA effects did not change with age [*r* = 0.05, *t*(187) = 0.70, NS].

Subjects were slightly faster to respond to stimuli in the visual field contralateral to the responding hand [by 2.6 ms, *F*(1,188) = 9.36, *p* < 0.003, ω^2^ = 0.04]. The average difference between SRT latencies in the left and right hemifield remained small (3.1 ms) and was not significantly correlated with age [*r* = 0.06, *t*(187) = 0.82, NS]. The absolute value of intrasubject differences in SRTs to stimuli in the left and right visual fields also remained small, although it was slightly larger than the differences seen in Experiment 1 [10.5 vs. 7.7 ms, *F*(1,1656) = 25.59, *p* < 0.0001, ω^2^ = 0.01].

**Figure 4** shows SDTs from individual subjects in the two experiments. SDTs were 7 ms longer in Experiment 2 than Experiment 1 [*F*(1,1656) = 8.97, *p* < 0.003, ω^2^ < 0.01 ]. As in Experiment 1, SDT latencies did not change significantly as a function of age [*r* = -0.05, NS]. In contrast, MITs increased with age [*r* = 0.43 (range 0.30–0.56), *t*(187) = 6.51, *p* < 0.0001] and, as in Experiment 1, showed a greater correlation with age than SRTs, although the difference in correlation coefficients did not reach significance [*z* = 0.91, *p* < 0.19].

There were several other small but significant differences between Experiment 1 and Experiment 2. Overall, higher values were seen in Experiment 2 for intrasubject SRT latency variance [*F*(1,1656) = 114.21, *p* < 0.0001, ω^2^ = 0.06] and CV [*F*(1,1656) = 132.49, *p* < 0.0001, ω^2^ = 0.07]. There were also two salient differences in the correlation matrices of the two experiments. Hit-rate increased with age in Experiment 1 [*r* = 0.17, *z* = 4.68, *p* < 0.0001], but declined with age in Experiment 2 [*r* = -0.19, *t*(187) = 2.65, *p* < 0.005]. Moreover, hit-rate was not significantly correlated with SRT latencies in Experiment 2 [*r* = -0.07, NS], unlike the apparent speed/accuracy trade-off [*r* = 0.25] seen in Experiment 1.

## Discussion: Experiment 2

Experiment 2 replicated the results of Experiment 1: mean SRTs differed by only 7 ms (230.8 vs. 237.8 ms) between the two studies, and by less than 10 ms when SRT latencies were corrected for age. The population standard deviations (28 vs. 27 ms) were also very similar in the two studies. The small difference in mean SRTs in the two experiments likely reflected the slightly increased mean and dispersion of SOAs in Experiment 2 (Niemi and Naatanen, 1981). Increased SOA dispersion may also have contributed to the increase in Experiment 2 intrasubject SRT variability and CV, which may also have been inflated due to the slight reduction in the number of test trials.

Highly significant age-related SRT slowing was also seen in both studies, with correlation coefficients of moderate magnitude [*r* = 0.24 and *r* = 0.35] and similar slopes (0.55 ms/year and 0.45 ms/year). In both experiments, SDT latencies did not change systematically with age. Since the MIT was estimated from a separate finger tapping task, any changes in the SRT latencies would be included in the SDT, i.e., the SRT-MIT difference. Therefore, the SDT would be expected to increase with SOA manipulations that prolong SRTs (Anstey et al., 2005). In both experiments, MITs showed greater correlations with age than did SRTs. Other factors, including sex and education, had minimal effects on performance in either experiment. Finally, the preceding SOA had a strong influence on SRT latencies in both experiments (effect sizes of 0.49 and 0.40), with SRTs being prolonged by 28 and 27 ms, respectively.

Importantly, SRT latencies across the two experiments were more consistent than in previous large-scale SRT replications, with the small differences in SRT latencies largely explained by the change in the range of SOAs. Other experiments, using apparently similar SRT paradigms and apparatus, have obtained more disparate results. For example, using a similar paradigm, Deary and Der (2005) reported mean SRTs of 328 ms, Deary et al. (2001) reported mean SRTs of 358 ms, Deary et al. (2011) reported mean SRTs of 256 ms, and Dykiert et al. (2012b) reported mean SRTs of 275 ms. As seen in **Table 1**, the mean SRTs from Deary et al. (2001) were roughly 2.7 SDs above the mean SRTs collected by Dykiert et al. (2012b). SRT latency replicability was better in studies with the ANAM military test battery. In the largest study, Vincent et al. (2012) reported SRTs of 261 ms, while Reeves et al. (2006) tested a population with a slightly lower mean age and reported SRTs of 285 ms; the SRT latency differences between these two studies, 24 ms, was more than three times the differences that we observed, and was accompanied by relatively large differences in both population standard deviations and age/SRT slopes. In contrast, the differences in SRTs between Experiment 1 and Experiment 2 were small in both absolute and age-corrected magnitude, and both experiments produced similar population SDs and age/SRT slopes. This suggests that the use of high-precision computer hardware and software can improve the precision and replicability of SRT latency measures.

## General Discussion

Delay-corrected SRT latencies were substantially shorter (213 and 220 ms in Experiments 1 and 2, respectively) than the SRT latencies reported in other large-scale computer-based studies, but were similar to the SRT latencies reported both by Galton (Johnson et al., 1985) and contemporary researchers using non-computerized measures (Eckner et al., 2011; Montare, 2013). Thus, unlike Woodley et al. (2013), we found no evidence of slowed processing speed in the contemporary populations that we tested.

The origin of the large variations in SRT latencies seen in recent computerized studies remains obscure. Differences in computer hardware and software (Plant and Turner, 2009) can, in some circumstances, add up to 100 ms to mean SRT latency measurements (Neath et al., 2011), and software interruptions could also increase latency measures and trial-to-trial SRT latency variability. In addition, visual SRTs are influenced by the SRT windowing function, the temporal pattern of stimulus delivery, stimulus luminance and contrast, and by the force and distance needed to activate the response button. Many of these variables have not been reported in previous SRT studies, and may have differed in experiments that used apparently similar methods.

### The Effects of Age on SRT Latencies

We found highly significant correlations of moderate magnitude between age and SRT latencies in both studies. The rate of age-related SRT slowing that we observed (mean 0.50 ms/year) was similar to that of Dykiert et al. (2012a), but considerably less than the slowing observed in other SRT studies (Deary and Der, 2005; Reeves et al., 2007; Vincent et al., 2008; Deary et al., 2011). We also found significant age-related increases in SRT variance, and, in Experiment 2, increases in the CV as well, consistent with previous reports (Dykiert et al., 2012a).

The nature of aging effects was further clarified by analyzing SDT latencies, which were not influenced by age in either study. This suggests that the age-related slowing of SRTs was largely due to the additional time required by older subjects to depress the response button, a hypothesis supported by stronger correlations observed in both studies between age and the MIT than between age and SRT latencies.

### Sex Differences

Sex differences have been found in mean SRTs and intrasubject SRT variability in a number of previous studies (Deary et al., 2001; Der and Deary, 2006; Dykiert et al., 2012b). In contrast, we found no sex differences in SRT latencies, nor did we find significant sex differences in intrasubject reaction time variance in either experiment. In this regard, Silverman (2006) reviewed studies of sex differences in SRTs performed throughout the 20th century. He found that the magnitude of reported sex differences declined markedly by the late 20th century, and speculated that reduced sex differences reflected increased opportunities for female participation in fast-action sports and driving.

## Conclusion

When measured with high-precision computer hardware and software, SRTs were obtained with short latencies (ca. 235 ms) that were similar across two large subject populations. When corrected for hardware and software delays, SRT latencies in young subjects were similar to those estimated from Galton’s historical studies, and provided no evidence of slowed processing speed in modern populations. SRTs to lateralized stimuli had slightly shorter latencies when the stimuli were presented in the visual field contralateral to the responding hand. However, the latency differences (<3 ms) were smaller than the delays expected from transcallosal transmission. SRTs increased with age at a rate of ∼0.5 ms/year, but were not significantly influenced by education or sex. The latency of stimulus detection, estimated from the difference in SRTs and movement initiation times measured in a finger tapping task, was stable across adulthood, suggesting that the age-related slowing of SRTs primarily reflected slowed motor output.

## Conflict of Interest Statement

David L. Woods is affiliated with NeuroBehavioral Systems, Inc., the developers of Presentation software used to create these experiments.

## Footnotes

1 http://www.ebire.org/hcnlab/cognitive-tests/SRT

2 Experiment 1 data was gathered as part of a study investigating the health effects of chronic exposure to low-level H_2_S (Reed et al., 2014), which revealed small but significant beneficial effects of H_2_S, such that persons in the highest two quartiles of exposure had SRTs that were 1.8 ms faster than persons in the lowest exposure quartile. Since age was weakly correlated with exposure (r = 0.035), the age regression reported here may slightly underestimate the effect of age on SRT in non-H_2_S exposed populations.

3 The CCAB included the following computerized tests and questionnaires: finger tapping, simple reaction time, Stroop, digit span forward and backward, phonemic and semantic verbal fluency, verbal list learning, spatial span, trail making, vocabulary, design fluency, the Wechsler Test of Adult Reading (WTAR), visual feature conjunction, risk and loss avoidance, delay discounting, the Paced Auditory Serial Addition Task (PASAT), the Cognitive Failures Questionnaire (CFQ), the Posttraumatic Stress Disorder Checklist (PCL), and a traumatic brain injury (TBI) questionnaire.
